# Supplementary figures and images for: Brain organoid formation on decellularized porcine brain ECM hydrogels
Source: PLoS One. 2021 Jan 28;16(1):e0245685. doi: 10.1371/journal.pone.0245685 (PMC7842896; doi:10.1371/journal.pone.0245685)

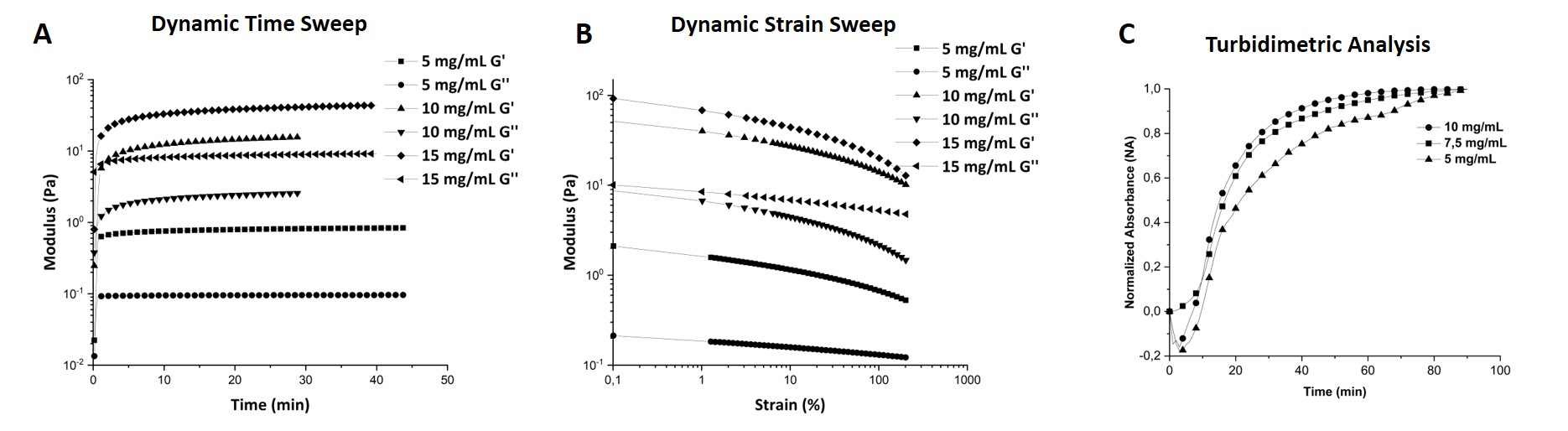

Supplement: S1 Fig — Rheological measurements were performed on B-ECM at concentrations of 5, 10 and 15 mg/mL with an ARES-LS2 Shear Rheometer (n = 3) (A) Dynamic time sweep analysis of hydrogel at different concentrations, showing storage modulus (G’) and loss modulus (G”) after initiation of gelation. (B) Strain sweep shows the physical failure of the gel at increased strain values between 0.1% to 200%. Gelation kinetics were furthermore observed by turbidimetric analysis. (C) Result of turbidimetric analysis for B-ECM hydrogels at concentrations of 5, 7.5 and 10 mg/mL (n = 6) at 37°C, measured at 405 nm in a photo spectrometer. (TIF) [file pone.0245685.s001.tif]

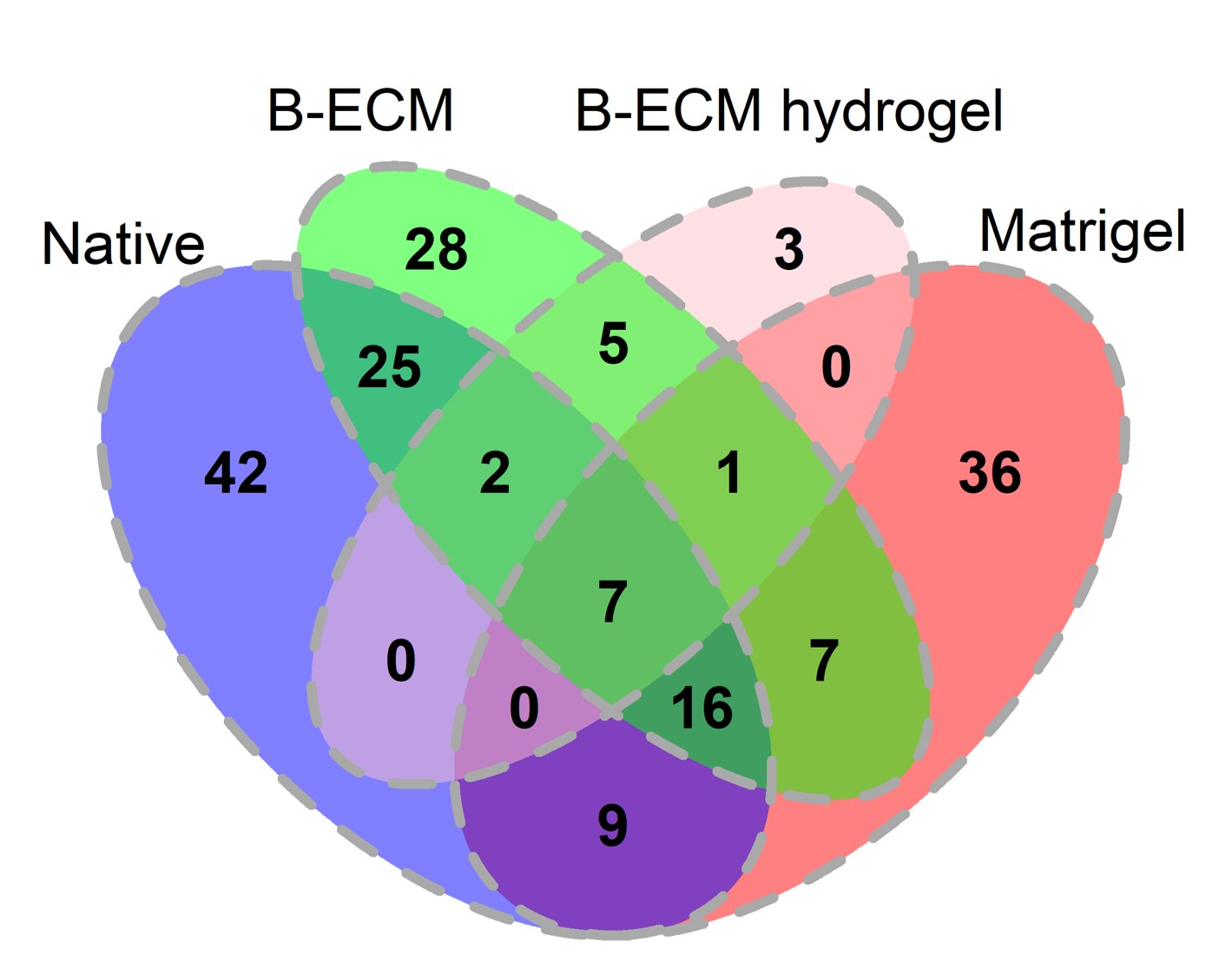

Supplement: S2 Fig — A Vonn-diagramm, showing the overlapping proteins per group which were detected with mass spectrometry. (TIF) [file pone.0245685.s002.tif]
