# Supplementary material for: Brain organoid formation on decellularized porcine brain ECM hydrogels
Source: PLoS One. 2021 Jan 28;16(1):e0245685. doi: 10.1371/journal.pone.0245685 (PMC7842896; doi:10.1371/journal.pone.0245685)
Supplement: S1 Table — Fw = forward, Rv = reverse. (DOCX) [file pone.0245685.s007.docx]

**S1 Table: Primers used for the RT-qPCR experiment. Fw = forward, Rv = reverse.**

| **Gene** | **Primer** | **Sequence 5´- 3´** | **Amplicon Size** |
| --- | --- | --- | --- |
| **NES** | Fw | ATCCTCAGTGGGTCAGACGA | 65 bp |
|  | Rv | CCCACATCTGAAACGACTCC |  |
| **TUBB3** | Fw | GCAACTACGTGGGCGACT | 85 bp |
|  | Rv | CGAGGCACGTACTTGTGAGA |  |
| **DCX** | Fw | GATGAATGGGTTGCCTAGCC | 66 bp |
|  | Rv | TGCAAGGTTCTGGTTCGGTAG |  |
| **MAP2** | Fw | GCTGACCTCAGCTGACAGAGA | 78 bp |
|  | Rv | GCTACAGCCTCAGCAGTGACTA |  |
| **GFAP** | Fw | ATCAACTCACCGCCAACAG | 61 bp |
|  | Rv | AGGTCCTGTGCCAGATTGTC |  |
| **ATP1B1** | Fw | TTAGGTTCCTGGAAAAGTACAAAGA | 95 bp |
|  | Rv | TCCTCGTTCTTTCGGTTCAC |  |
| **18S** | Fw | ATCCATTGGAGGGCAAGTC | 106 bp |
|  | Rv | GCTCCCAAGATCCAACTACG |  |
| **TBP (2)** | Fw | GAACATCATGGATCAGAACAACA | 87 bp |
|  | Rv | ATAGGGATTCCGGGAGTCAT |  |
| **HPRT** | Fw | TGACCTTGATTTATTTTGCATACC | 102 bp |
|  | Rv | CGAGCAAGACGTTCAGTCCT |  |
