# Supplementary material for: Brain organoid formation on decellularized porcine brain ECM hydrogels
Source: PLoS One. 2021 Jan 28;16(1):e0245685. doi: 10.1371/journal.pone.0245685 (PMC7842896; doi:10.1371/journal.pone.0245685)
Supplement: Attachment — Submitted filename: Response to reviewers.docx [file pone.0245685.s008.docx]

November 22, 2020

Subject: Resubmission of manuscript PONE-D-20-23633

Dear Dr. Nic D. Leipzig and Reviewers,

Thank you for your review of the manuscript “Brain organoid formation on decellularized porcine brain ECM hydrogels” and your invitation to resubmit, following a thorough revision. Your comments have been very helpful for us and we have revised the manuscript accordingly.

We have included the reviewer comments immediately after this letter (presented in *Italic*) with our answers below each comment in dark blue for better visibility. When changes have been applied, line number in manuscript is given as “L Number”, and changed paragraph is copied below the answer within quotation marks in orange. When references were given, they were presented as Author+Year and given in more details at the end of this document in the “References Section”.

**Editors Decision:**

*PONE-D-19-11827
Effect of fluid dynamics on decellularization efficacy and mechanical properties of blood vessels
PLOS ONE

Dear Dr. Simsa,*

*Thank you for submitting your manuscript to PLOS ONE. After careful consideration, we feel that it has merit but does not fully meet PLOS ONE’s publication criteria as it currently stands. Therefore, we invite you to submit a revised version of the manuscript that addresses the points raised during the review process.*

*Please submit your revised manuscript by Nov 23 2020 11:59PM. If you will need more time than this to complete your revisions, please reply to this message or contact the journal office at plosone@plos.org. When you're ready to submit your revision, log on to https://www.editorialmanager.com/pone/ and select the 'Submissions Needing Revision' folder to locate your manuscript file.*

*Please include the following items when submitting your revised manuscript:*

*•	A rebuttal letter that responds to each point raised by the academic editor and reviewer(s). You should upload this letter as a separate file labeled 'Response to Reviewers'.*

*•	A marked-up copy of your manuscript that highlights changes made to the original version. You should upload this as a separate file labeled 'Revised Manuscript with Track Changes'.*

*•	An unmarked version of your revised paper without tracked changes. You should upload this as a separate file labeled 'Manuscript'.*

*If you would like to make changes to your financial disclosure, please include your updated statement in your cover letter. Guidelines for resubmitting your figure files are available below the reviewer comments at the end of this letter.*

*If applicable, we recommend that you deposit your laboratory protocols in protocols.io to enhance the reproducibility of your results. Protocols.io assigns your protocol its own identifier (DOI) so that it can be cited independently in the future. For instructions see: http://journals.plos.org/plosone/s/submission-guidelines#loc-laboratory-protocols*

*We look forward to receiving your revised manuscript.*

*Kind regards,*

*Nic D. Leipzig*

*Academic Editor, PLOS ONE*

**Journal Requirements:**

*When submitting your revision, we need you to address these additional requirements.*

*1. Please ensure that your manuscript meets PLOS ONE's style requirements, including those for file naming. The PLOS ONE style templates can be found at*

*https://journals.plos.org/plosone/s/file?id=wjVg/PLOSOne_formatting_sample_main_body.pdf and*

*https://journals.plos.org/plosone/s/file?id=ba62/PLOSOne_formatting_sample_title_authors_affiliations.pdf*

*2. We understand that you purchased animal tissue from Amazon.com for this study. In your Methods section, please provide additional regarding the source of this material, specifically any details about the purchased items (e.g., lot number, source origin, description of appearance) that will facilitate reproducibility of the analyses.*

Thank you for your comment. We have added a more detailed description to the methods section of the manuscript (L 101 – 103):

“Frozen whole pig brains (FarmLand, USA) were obtained via Amazon.com and stored at -20°C. Brains were derived from adult animals (6 months at time of slaughter), mixed gender, of Smithfield breed, raised in the USA. Brains were delivered in a sealed plastic cup without any additives or preservatives.”

*3. Thank you for stating the following in the Acknowledgments Section of your manuscript:*

*"This project has received funding from the European Union’s Horizon 2020 research and innovation 594 program under the Marie Skłodowska-Curie grant agreement No 722779 and was conducted within the 595 “Training 4 Cell Regenerative Medicine” (T4CRM) network. Work at AMS group (CBMSO-UAM) was also 596 supported by grants MINECO SAF-2017-83241-R, and ISC-III RETICS TerCel RD16/0011/0032 and ASCTN597 Training (No. 813851). Work at Tufts was supported by the NIH (P41EB027062 and R01NS092847)".*

i) *We note that you have provided funding information that is not currently declared in your Funding Statement. However, funding information should not appear in the Acknowledgments section or other areas of your manuscript. We will only publish funding information present in the Funding Statement section of the online submission form.*

*ii) Please remove any funding-related text from the manuscript and let us know how you would like to update your Funding Statement. Currently, your Funding Statement reads as follows:*

*"The funders had no role in study design, data collection and analysis, decision to publish, or preparation of the manuscript."*

** Please include your amended statements within your cover letter; we will change the online submission form on your behalf.*

We have deleted the funding statement from the main manuscript and added it to the online submission of PLOS ONE in the field “Funding Information”. Also, we added this information to the cover letter.

*4. Please include captions for your Supporting Information files at the end of your manuscript, and update any in-text citations to match accordingly. Please see our Supporting Information guidelines for more information: http://journals.plos.org/plosone/s/supporting-information.*

Supporting information files have previously been uploaded as an individual file, but they were added to the end of the manuscript (after the references) in this version (starting from L-821).

*Additional Editor Comments (if provided):*

*Also, to add to the reviewers comments, my biggest question with this work and the materials used is do the authors include the dura and the pia in the harvested brains and does that ECM carry over the matrix that is created form them? The reason I mention this is that the parenchyma of the CNS is well documented to contain very little matrix that is almost devoid of fibrillary matrix (especially fibrillary collagens), and it is mostly cells by volume (nearly 75% by some reports) (e.g., Zimmermann, 2008, DOI: 10.1007/s00418-008-0485-9). Thus the B-ECM hydrogels used are not necessarily what the authors think they are, and definitely not what they are being represented as. To me they are a mixed up version of the adult pig native brain and its associated basement membranes – I would expect nearly none of the collagens that Fig 3 proteomic analysis shows if purer white/grey matter was used. To be honest using a purer source would probably be hard to gel natively. So the bigger question in terms of the mixed up ECM nature of the B-ECM hydrogels is this a good or a bad thing in terms of desired application for brain organoids?*

Thank you for your comment. It was not our approach to separate the dura and pia mater from the parenchyma, therefore some explanation in the manuscript may have been misleading. In our case we reported a loss of 98.7 % weight, with a remaining ECM weight of less than 1.3%. The collagen content was 113 µg/mg dry weight. This is in line with studies performed by other groups on whole brain decellularization (Sood *et al.*, 2016; Koh *et al.*, 2018). Collagen is present at much lower levels in the brain parenchyma compared to other tissues (Ruoslahti, 1996), however the presence of different collagen types in the brain has also been reported in previous studies, partly originating from the microvasculature (Ogawa *et al.*, 1989; Kucharz and Kucharz, 1992; Payne and Huang, 2013; Gregorio *et al.*, 2018). We added a paragraph to the introduction and discussion to address the use of whole pig brain and the potential source of collagen from the dura and pia (L 92 – 93 and L 523 – 527):

“In the present study, we evaluated the use of decellularized whole pig brain ECM hydrogels as a scaffold for cerebral organoid growth, in comparison to the commonly applied Matrigel. “

“Collagen is less abundant in brain tissue compared to other tissue types [11], however different collagen types are present in parts of the brain such as the microvascular [50]. This may explain the remaining collagen content, which has also been reported in other studies performing brain DC [22].As whole pig brains were used in this study, also collagen from the dura mater and pia mater may be responsible for the observed collagen content [51].”

*My other point I would like to add is the data shown (beyond Fig.1) appears to be relatively devoid of statistical analyses.*

Some figures include macroscopic images without numerical data, however results for qPCR, collagen/GAG quantification and rheology were also analyzed statistically. We added another explanation to the legend of table 1 (L 425 – 427) and figure 5 (L and L 461 - 472):

“Table 1. Analysis of mechanical properties and gelation kinetics of B-ECM hydrogels at different concentrations. Statistical differences in turbidimetric analysis between different rows are indicated with an asterix (*).”

(C) Expression of the genes NEST, TUBB3, DCX, MAP2 and GFAP was measured by extraction of RNA from organoids grown in the presence of B-ECM hydrogel (n=13) or Matrigel (n=12) in technical triplicates with RT-qPCR. Statistical analysis performed with unpaired t-test.

*Please fully address all reviewer and my comments if you wish to resubmit.*

***Reviewer #1:***

*This is an interesting and well written paper. It would benefit from some key clarifications as there are multiple ECM endpoints examined that need better grouping, rationale and detail:*

*-Pig brains were decellularized, but appear to retain intravascular contents (ie they were not perfused) correct? This is a key point.*

Thank you for your comment. The pig brains were cut into small pieces before decellularization, therefor the whole content of the native brain was present. We have added a paragraph in the methods section to describe the decellularization process (L 103 – 104):

“For decellularization, the brain tissue was thawed, cut into small pieces with a scalpel, and added to a 1L glass bottle.”

Intravascular content (e.g. content found in blood) was likely removed in large part during the washing and Decellularization process. However, the occurrence of intravascular collagen content in the brain may be responsible for parts of the observed collagen content in the B-ECM hydrogel. We have added a paragraph to the discussion to reflect this observation (L 522 – 526):

“The B-ECM and B-ECM hydrogel group had a high abundance (greater than 90%) of collagens. Collagen is less abundant in brain tissue compared to other tissue types (Ruoslahti, 1996), however different collagen types are present in parts of the brain such as the microvascular (Kucharz and Kucharz, 1992). This may explain the remaining collagen content, which has also been reported in other studies performing brain DC (Sood *et al.*, 2016).”

*-Fig 1 collagen content should define which collagens are detected with the kit and whether that is relevant to brain.*

Thank you for your comment. For the quantification of collagen, the Sircol soluble collagen assay (Biocolor, United Kingdom) was used. This assay is able to detect collagens that are solubilized with the presented hydrogel preparation technique (based on pepsin-HCl), and are therefore relevant for the gelling properties of the hydrogel. Additional analysis of different collagen types was carried out in the Mass Spec analysis (Fig. 3). We have added the description of soluble collagen detection in the result section of the manuscript (L 328 – 330):

“Due to cell removal, the soluble collagen content was significantly increased in the decellularized sample (113.5 ± 24.65 µg/mg dry weight) compared to the native sample (14.11 ± 1.15 µg/mg dry weight) (Fig 1E).”

*-Similarly, GAGs measured with the kit used are only sulfated GAGs, this should be noted. Also was HA (nonsulfated GAG) measured? why not?*

To quantify GAGs, we utilized the glycosaminoglycan assay from Chondrex, which indeed only measures sulfated GAGs, which is a limitation of this assay. However, a more thorough analysis of different GAGs present (also nonsulfated) was performed in the Mass Spec analysis (Figure 3). To describe the properties of the GAG assay more precisely, we added this information to the methods section (L 170 – 171) and the results section (L 326 – 328):

“For GAG quantification, the glycosaminoglycan assay (Chondrex, USA) was used, which detects sulfated GAGs.”

“The content of sulfated GAGs was significantly decreased in the DC sample (0.70 ± 0.33 µg/mg wet tissue) compared to the native sample (1.71 ± 0.63 µg/mg wet tissue) (Fig 1D).”

*-Fig 2 the choices for IHC are not well justified. The authors should explain why those ECM molecules were chosen. If focusing on basement membrane, then why BMP and HLA-DR? Vitronectin seems out of place here.*

Thank you for your comment. While laminin and fibronectin are well established proteins of the brain ECM (Ruoslahti, 1996; Tonge *et al.*, 2012), also vitronectin has been shown to be present in the native brain and has been shown *in vitro* to promote neuronal survival and outgrowth (Grabham, Gallimore and Grand, 1992; Marti ́nez-Morales *et al.*, 1995), and has therefor been included in the IHC analysis. HLA-DR and MBP have been selected as markers of donor-specific immunogenicity. Also other markers could be used for this purpose, but HLA-DR and BMP have also previously been used for the evaluation of immunogenicity of decellularized tissues (Sackett *et al.*, 2018). If proposed by the reviewer, these marker proteins can also be deleted from the IHC figure.

*-Fig 3 is matrisome analysis, but should discuss the molecules based on their relevance and location in the brain.
-In addition to noting whether the ECM molecules of interest are relevant to vasculature (whether glycocalyx or basement membrane) or brain parenchyma ECM, a summary of the ECM findings in chart form might be helpful.*

Thank you for your comments. Fig 3 is indeed an analysis of the proteins detected in the B-ECM hydrogel group, in relation to the same proteins detected in the other tested groups (Native, B-ECM, Matrigel). While this figure intends to give a quick overview over the relevant protein groups detected, we have also added in-depth charts on the other conclusions of the matrisome analysis in the supplementary information with 4 files, where the function, location and overlapping proteins between different groups can be observed in detail:

Supplementary File 1: Raw data of the Mass Spectrometry results

Supplementary File 2: Normalized and filtered Mass Spectrometry results

Supplementary File 3: Normalized and filtered Mass Spectrometry results with analysis of overlapping detected proteins between different testing groups, corresponding to the Venn-Diagram

Supplementary File 4: Normalized and filtered Mass Spectrometry results, filtered for only matrisome proteins

These charts have been added to the supplementary information section in order to simplify the figures in the main manuscript, and can be downloaded in a compacted .zip file at the end of the manuscript. If proposed by the reviewer, some of this information may also be transferred to the main manuscript.

***Reviewer #2:***

*In this paper, Simsa and colleagues have introduced a decellularized porcine brain extracellular matrix (B-ECM) as an advanced alternative scaffold for human embryonic stem cell (hESC)-derived brain organoids. Compared to matrigel, which is conventionally used to provide structural support and biological cues for the brain organoid maturation, B-ECM is known to have more similar and abundant components of brain extracellular matrix. The authors first analyzed the properties of B-ECM hydrogels, including their composition, surface structure, and viscoelastic behavior, and compare with those of matrigel, showing that B-ECM is suitable as a biocompatible scaffold for brain organoid culture. Next, they embedded the cerebral organoids in B-ECM hydrogel and observed smoother neural epithelial development than those in matrigel, suggesting that B-ECM indeed provides substantial structural support for the growth of brain organoids. Although the authors well-presented analytical data on B-ECM hydrogel for brain organoid culture, their data is insufficient to argue that B-ECM provides more advantageous growth environment than matrigel. The results merely proved that the usage of B-ECM can yield the similar level of maturation process of brain organoids compared to matrigel.


Major comments

(1) If there was no significant differences observed during the maturation stage of organoid when either B-ECM or matrigel were used, why should researchers use B-ECM hydrogel rather than matrigel? Also, why does B-ECM induce smoother neural epithelial development compared to matrigel? These questions should be addressed in order to persuade the readers that B-ECM is more beneficial to use for brain organoid culture.*

Thank you for your comment. It is correct that we saw only minor differences in the culture of 3D brain organoids on Matrigel or on B-ECM hydrogels. At the current point, B-ECM hydrogels do not show to be a preferred alternative. However, we believe that optimization of B-ECM hydrogel preparation techniques can help to create more relevant and tissue-specific hydrogels for organoid generation compared to Matrigel. Our study is a first evaluation of B-ECM hydrogels for 3D brain organoid culture, but protocols need to be improved in further studies to support the retention of brain-specific proteins in the ECM and potentially lead to a more native-like behavior of the organoids. In our paper, we have concluded that it is still challenging to find an ideal balance between cell removal and preservation of ECM proteins. While we detected several proteins in the native brain and the B-ECM which are relevant for the brain (e.g. BCAN and VCAN), these proteins were not detected after hydrogel preperation in the B-ECM hydrogel group. This result shows that the current standard for hydrogel preparation, including pepsin-HCl, may be too harsh for the retention of brain specific proteins and GAGs. The development of additional hydrogel processing methods is seen as beneficial for more native-like decellularized hydrogels. To show the need for further optimization of B-ECM hydrogels, we have added following paragraph to the discussion (L 574 – 578):

“From these observations, we conclude that the B-ECM produced by this protocol supports the development of neuroepithelial tissue as well as Matrigel and can thus be considered as a suitable alternative hydrogel for developmental studies of the human brain. Further optimization of DC protocols to improve the retention of functional proteins could lead to more enhanced effects of applying B-ECM.”

*(2) The components of B-ECM is rather different than what authors initially mentioned. B-ECM is known to have more glycosaminoglycans (GAGs) and includes less portion of fibrous proteins, such as collagen. However, their analytical data showed that their B-ECM is highly enriched with collagen while the percentage of proteoglycans and glycoproteins is very low compared to matrigel. Indeed, in Figure 3A, matrigel has more ‘ideal’ composition rather than B-ECM hydrogel. Why is the author’s B-ECM possess such a low level of GAGs components?*

Thank you for your comment. It is true that the GAG content in the native brain is reported to be higher compared to collagen (Ruoslahti, 1996; Lau *et al.*, 2013), and we detected a lower number of GAGs in our B-ECM hydrogel compared to the native brain (Fig 3). We observed that there was a loss of GAGs as a result of the decellularization process, which led to decreased content of GAGS in the B-ECM and the B-ECM hydrogel, as observed with Mass Spec analysis.

We also observed that the brain is an especially challenging tissue for decellularization, as methods which we previously effectively applied for other tissues (e.g. blood vessels or nerves) did not lead to a complete removal of cellular material. Therefore, we had to use harsher decellularization methods, which may have contributed to a removal of GAGs. The removal of GAGs however, is also an expected side effect of the tissue decellularization, which is reported for all tissues decellularized with detergent/enzyme based methods and also reflects the removal of cellular GAGs during DC (Badylak, Taylor and Uygun, 2011; White *et al.*, 2017a). Improved monitoring techniques may help to further improve decellularization protocols in the future (Geerts *et al.*, 2016). To address the GAG removal, we have clarified our findings in the discussion (L 545 - 549):

“Loss of GAGs in the DC process may in part be explained by the removal of cells, which contain membrane–bound GAGs. Removal of GAGs is seen as unbeneficial, as GAGs can guide cell behavior (Ushakova *et al.*, 2002). However, GAG removal is a common result of detergent/enzyme based DC, and has been observed in multiple different studies (GILBERT, SELLARO and BADYLAK, 2006; Badylak, Taylor and Uygun, 2011; Keane, Swinehart and Badylak, 2015; White *et al.*, 2017b). Improvement of the DC protocol may lead to a better retention of GAGs in the decellularized tissue (Geerts *et al.*, 2016).”

*(3) They introduced the ‘novel’ methodology to synthesize a decellularized B-ECM. How is the novel method more advantageous over the previous methods? More details on the methodologies and comparison with others should be addressed in order to persuade the readers that the methods described here perform well.*

Thank you for your comment. While other protocols often use a larger number of different detergent/enzymes, we simplified the DC process by only applying a single detergent and a single enzymatic agent. Using less detergent is advantageous for the evaluation of DC parameters, which can be linked to only one detergents compared with interfering effects of multiple different detergents. Furthermore, several protocols apply SDS, which has previously been shown to damage ECM proteins and lead to collagen hybridization. We have added another paragraph to our discussion for a clarification of our rationale (L 490 -498):

“Initially, we tested different DC protocols based on combinations of SDS, TritonX or SDC as well as DNA removing enzymes. From these initial experiments we developed a new protocol based on the ionic detergent SDC and DNase. SDC proved to be a suitable DC detergent for the large brain volume, with little or no cellular remnants or antigens detectable after DC. The use of SDC-based protocols has previously shown to be beneficial over SDS-based protocols, as increased damage to ECM proteins in different tissue DC applications has been reported with SDS (Faulk *et al.*, 2014; Poornejad *et al.*, 2016; Hwang *et al.*, 2017). Another advantage of this protocol is that it only applies a single detergent and a single enzyme, which simplifies evaluation and optimization of the protocol compared to protocols using multiple different detergents for DC (Crapo *et al.*, 2012; Sood *et al.*, 2016; Lin *et al.*, 2017).” *Minor comments

(1) - There is no information about the pig’s brain described such as age, sex, the region of the brain, and presence of inflammation. There might be some difference in ECM composition between young and old pig brains.*

We have updated the methods section of the manuscript to give more information on the pig brains origin (L 101-102).

“Frozen whole pig brains (FarmLand, USA) were obtained via Amazon.com and stored at -20°C. Brains were derived from adult animals (6 months at time of slaughter), mixed gender, of Smithfield breed, raised in the USA. Brains were delivered in a sealed plastic cup without any additives or preservatives.”

*(2) The spelling or typos must be checked. For example, in line 546, ‘therefor’ should be changed to ‘therefore.’*

Thank you for your comment. We have checked spelling errors with a specialized software, and applied appropriate changes.

*(3) In figure 4, there are no letter labels (A, B, C). Please check the page 10, line 365 and 369.*

Thank you for your thorough attention on our manuscript. Indeed, there was a numbering error, as we intended to refer to “Figure 3”, while “Figure 4” was a mistake. We have edited the manuscript to cite the correct figure 3 now.

*(4) In page 14, “DNAse” -> “DNase”*

Thank you for your comment, we have corrected the spelling which reads “DNase” now (L 492)

**References:**

Badylak, S. F., Taylor, D. and Uygun, K. (2011) ‘Whole-Organ Tissue Engineering: Decellularization and Recellularization of Three-Dimensional Matrix Scaffolds’, *Annual Review of Biomedical Engineering*, 13(1), pp. 27–53. doi: 10.1146/annurev-bioeng-071910-124743.

Crapo, P. M. *et al.* (2012) ‘Biologic scaffolds composed of central nervous system extracellular matrix’, *Biomaterials*. NIH Public Access, 33(13), p. 3539. doi: 10.1016/J.BIOMATERIALS.2012.01.044.

Faulk, D. M. *et al.* (2014) ‘The effect of detergents on the basement membrane complex of a biologic scaffold material.’, *Acta biomaterialia*. NIH Public Access, 10(1), pp. 183–93. doi: 10.1016/j.actbio.2013.09.006.

Geerts, S. *et al.* (2016) ‘Nondestructive Methods for Monitoring Cell Removal During Rat Liver Decellularization’, *Tissue Engineering - Part C: Methods*. Mary Ann Liebert Inc., 22(7), pp. 671–678. doi: 10.1089/ten.tec.2015.0571.

GILBERT, T., SELLARO, T. and BADYLAK, S. (2006) ‘Decellularization of tissues and organs’, *Biomaterials*. doi: 10.1016/j.biomaterials.2006.02.014.

Grabham, P. W., Gallimore, P. H. and Grand, R. J. A. (1992) ‘Vitronectin is the major serum protein essential for NGF-mediated neurite outgrowth from PC12 cells’, *Experimental Cell Research*. Academic Press, 202(2), pp. 337–344. doi: 10.1016/0014-4827(92)90083-K.

Gregorio, I. *et al.* (2018) ‘Collagen VI in healthy and diseased nervous system’, *DMM Disease Models and Mechanisms*. Company of Biologists Ltd. doi: 10.1242/dmm.032946.

Hwang, J. *et al.* (2017) ‘Molecular assessment of collagen denaturation in decellularized tissues using a collagen hybridizing peptide’, *Acta Biomaterialia*, 53, pp. 268–278. doi: 10.1016/j.actbio.2017.01.079.

Keane, T. J., Swinehart, I. T. and Badylak, S. F. (2015) ‘Methods of tissue decellularization used for preparation of biologic scaffolds and in vivo relevance’, *Methods*. Academic Press, 84, pp. 25–34. doi: 10.1016/J.YMETH.2015.03.005.

Koh, I. *et al.* (2018) ‘The mode and dynamics of glioblastoma cell invasion into a decellularized tissue-derived extracellular matrix-based three-dimensional tumor model’, *Scientific Reports*, 8(1), p. 4608. doi: 10.1038/s41598-018-22681-3.

Kucharz, E. J. and Kucharz, E. J. (1992) ‘Collagen in the Nervous System’, in *The Collagens: Biochemistry and Pathophysiology*. Springer Berlin Heidelberg, pp. 261–263. doi: 10.1007/978-3-642-76197-3_19.

Lau, L. W. *et al.* (2013) ‘Pathophysiology of the brain extracellular matrix: A new target for remyelination’, *Nature Reviews Neuroscience*. Nature Publishing Group, pp. 722–729. doi: 10.1038/nrn3550.

Lin, Q. *et al.* (2017) ‘Enhanced neuroprotection with decellularized brain extracellular matrix containing bFGF after intracerebral transplantation in Parkinson’s disease rat model’, *International Journal of Pharmaceutics*, 517(1–2), pp. 383–394. doi: 10.1016/j.ijpharm.2016.12.028.

Marti ́nez-Morales, J. R. *et al.* (1995) ‘Developmentally regulated vitronectin influences cell differentiation, neuron survival and process outgrowth in the developing chicken retina’, *Neuroscience*. Neuroscience, 68(1), pp. 245–253. doi: 10.1016/0306-4522(95)00089-2.

Medberry, C. J. *et al.* (2013) ‘Hydrogels derived from central nervous system extracellular matrix.’, *Biomaterials*. NIH Public Access, 34(4), pp. 1033–40. doi: 10.1016/j.biomaterials.2012.10.062.

Ogawa, K. *et al.* (1989) ‘Distribution of collagen Type IV in brain tumors: An immunohistochemical study’, *Journal of Neuro-Oncology*. Kluwer Academic Publishers, 7(4), pp. 357–366. doi: 10.1007/BF02147093.

Payne, L. S. and Huang, P. H. (2013) ‘The pathobiology of collagens in glioma’, *Molecular Cancer Research*. American Association for Cancer Research Inc., pp. 1129–1140. doi: 10.1158/1541-7786.MCR-13-0236.

Poornejad, N. *et al.* (2016) ‘The impact of decellularization agents on renal tissue extracellular matrix’, *Journal of Biomaterials Applications*, 31(4), pp. 521–533. doi: 10.1177/0885328216656099.

Ruoslahti, E. (1996) ‘Brain extracellular matrix.’, *Glycobiology*, 6(5), pp. 489–92. Available at: http://www.ncbi.nlm.nih.gov/pubmed/8877368 (Accessed: 30 April 2019).

Sackett, S. D. *et al.* (2018) ‘Extracellular matrix scaffold and hydrogel derived from decellularized and delipidized human pancreas’, *Scientific Reports*. Nature Publishing Group, 8(1), p. 10452. doi: 10.1038/s41598-018-28857-1.

Sood, D. *et al.* (2016) ‘Fetal brain extracellular matrix boosts neuronal network formation in 3D bioengineered model of cortical brain tissue.’, *ACS biomaterials science & engineering*. NIH Public Access, 2(1), pp. 131–140. doi: 10.1021/acsbiomaterials.5b00446.

Tonge, D. A. *et al.* (2012) ‘Fibronectin supports neurite outgrowth and axonal regeneration of adult brain neurons in vitro.’, *Brain research*. Elsevier, 1453(100), pp. 8–16. doi: 10.1016/j.brainres.2012.03.024.

Ushakova, G. A. *et al.* (2002) ‘Extracellular matrix heparin induces alteration of the cell adhesion during brain development.’, *Neurochemistry international*, 40(3), pp. 277–83. Available at: http://www.ncbi.nlm.nih.gov/pubmed/11741012 (Accessed: 9 May 2019).

White, L. J. *et al.* (2017a) ‘The impact of detergents on the tissue decellularization process: A ToF-SIMS study.’, *Acta biomaterialia*. NIH Public Access, 50, pp. 207–219. doi: 10.1016/j.actbio.2016.12.033.

White, L. J. *et al.* (2017b) ‘The impact of detergents on the tissue decellularization process: A ToF-SIMS study.’, *Acta biomaterialia*. NIH Public Access, 50, pp. 207–219. doi: 10.1016/j.actbio.2016.12.033.
