# Supplementary material for: Brain organoid formation on decellularized porcine brain ECM hydrogels
Source: PLoS One. 2021 Jan 28;16(1):e0245685. doi: 10.1371/journal.pone.0245685 (PMC7842896; doi:10.1371/journal.pone.0245685)
Supplement: Attachment — Submitted filename: Response to reviewers.docx [file pone.0245685.s009.docx]

December 25, 2020

Subject: Resubmission of manuscript PONE-D-20-23633

Dear Dr. Nic D. Leipzig and Reviewers,

Thank you for your further review of the manuscript “Brain organoid formation on decellularized porcine brain ECM hydrogels” and your invitation to resubmit. Your comments have been very helpful to further improve the clarity of the manuscript and we have adopted them accordingly.

We have included the reviewer comments immediately after this letter (presented in *Italic*) with our answers below each comment in dark blue for better visibility. When changes have been applied, line number in manuscript is given as “L Number”, and changed paragraph is copied below the answer within quotation marks in orange.

**Editors Decision:**

*PONE-D-20-23633R1
Brain organoid formation on decellularized porcine brain ECM hydrogels
PLOS ONE

Dear Dr. Simsa,

Thank you for submitting your manuscript to PLOS ONE. After careful consideration, we feel that it has merit but does not fully meet PLOS ONE’s publication criteria as it currently stands. Therefore, we invite you to submit a revised version of the manuscript that addresses the points raised during the review process.

Thank  you for the revision, overall the manuscript is much improved.  Reviewer 1 brings up some important points I agree with - namely making sure that the abstract and the conclusion convey the importance of collagen in the B-ECM treatments in this study. I will add an additional concern about this as well, namely that an adult ECM is being used in an attempt to drive an early embryonic process. We all hate Matrigel, but its tumorigenic-derived composition may actually be closer to an early embryonic matrix composition then any decellularized (DC) adult tissue. The last paragraph of the discussion kind of points this out, but I think itneeds to be more explicit in terms of brain organoids and their significant developmental timeline differences from the DC tissues used.

Please submit your revised manuscript by Feb 07 2021 11:59PM. If you will need more time than this to complete your revisions, please reply to this message or contact the journal office at* [*plosone@plos.org*](mailto:plosone@plos.org)*. When you're ready to submit your revision, log on to* [*https://www.editorialmanager.com/pone/*](https://www.editorialmanager.com/pone/) *and select the 'Submissions Needing Revision' folder to locate your manuscript file.

Please include the following items when submitting your revised manuscript:*

- *A rebuttal letter that responds to each point raised by the academic editor and reviewer(s). You should upload this letter as a separate file labeled 'Response to Reviewers'.*
- *A marked-up copy of your manuscript that highlights changes made to the original version. You should upload this as a separate file labeled 'Revised Manuscript with Track Changes'.*
- *An unmarked version of your revised paper without tracked changes. You should upload this as a separate file labeled 'Manuscript'.*

*If you would like to make changes to your financial disclosure, please include your updated statement in your cover letter. Guidelines for resubmitting your figure files are available below the reviewer comments at the end of this letter.

If applicable, we recommend that you deposit your laboratory protocols in protocols.io to enhance the reproducibility of your results. Protocols.io assigns your protocol its own identifier (DOI) so that it can be cited independently in the future. For instructions see:* [*http://journals.plos.org/plosone/s/submission-guidelines#loc-laboratory-protocols*](http://journals.plos.org/plosone/s/submission-guidelines#loc-laboratory-protocols) *We look forward to receiving your revised manuscript.

Kind regards,

Nic D. Leipzig
Academic Editor
PLOS ONE


Additional Editor Comments (if provided):

Thank you for the revision, overall the manuscript is much improved. Reviewer 1 brings up some important points I agree with - namely making sure that the abstract and the conclusion convey the importance of collagen in the B-ECM treatments in this study. I will add an additional concern about this as well, namely that an adult ECM is being used in an attempt to drive an early embryonic process. We all hate Matrigel, but its tumorigenic-derived composition may actually be closer to an early embryonic matrix composition then any decellularized adult tissue. The last paragraph of the discussion kind of points this out, but I think needs to be more explicit in terms of brain organoids and their significant developmental timeline differences from the DC tissues used.*

Thank you for your suggestions. We share your view that the matter of remaining collagen as well as the use (and limitations) of using adult pig brains should be highlighted. We therefor first included a clarification in the abstract (L 25-28, L 32-34 and L 36-39):

“In this study, we investigated the application of a decellularized adult porcine brain extracellular matrix (B-ECM) which could be processed into a hydrogel (B-ECM hydrogel) to be used as a scaffold for human embryonic stem cell (hESC)-derived brain organoids.”

“We found that the native brain source material was successfully decellularized with little remaining DNA content, while Mass Spectrometry (MS) showed the loss of several brain-specific proteins, while mainly different collagen types remained in the B-ECM.”

“These results indicate that B-ECM hydrogels can be used as an alternative scaffold for human cerebral organoid formation, and may be further optimized for improved organoid growth by further improving protein retention other than collagen after decellularization.”

Furthermore, we have highlighted the reasoning and limitations of utilizing adult pig brains in the discussion (L 584-590):

“Especially the age of the animal could affect neuronal network formation, as the ECM is dynamically remodeled and shows a different protein composition at different life stages [73], as previously shown in a comparison of fetal and adult pig brain ECM [22]. Also in this study, adult pig brains were used, due to good availability, cheap costs and the practical and economical difficulties of utilizing embryonic pig brains. Indeed, embryonic pig brains may be a more suitable model for brain organoid development [22], however the high costs of obtaining embryonic pig brains and ethical concerns may be a practical hurdle for large scale adoption as a model for brain organoid development compared to Matrigel.”

Also, we clarified the use of adult pig brains and the remaining collagen content in the conclusion (L 595-601):

“In this study, we showed that porcine B-ECM hydrogels could be produced from native adult porcine brain, with few host cells remaining. During DC and hydrogel processing, several functional proteins were lost, however, this did not negatively impact organoid utility. After DC, the B-ECM consisted mainly of different collagen types, which is beneficial in terms of hydrogel stability, but may not have the same beneficial functional effect as if more functional, brain-specific proteins were retained. Hence, the process described here can be further optimized to reduce extraction losses and increase the content of functional proteins in the hydrogel.”


***Reviewer comments:***

*Reviewer #1: The authors did a commendable job of addressing concerns. However, given the dominance of collagen in the final product, the manuscript needs to be revised to reflect this. At present there are just side references to this collagen-dominant composition in the results and discussion with little attention to the implications for utilization of the organoid model. The abstract and conclusion do not even mention the collagens. A different perspective in the writing is warranted.*

Thank you for your comments. We have adopted the manuscript to highlight the remaining collagen after DC also in the abstract and the conclusion of the manuscript. We have changed following paragraph in the abstract (L 32-34 and L 36-39):

“We found that the native brain source material was successfully decellularized with little remaining DNA content, while Mass Spectrometry (MS) showed the loss of several brain-specific proteins, while mainly different collagen types remained in the B-ECM.”

“These results indicate that B-ECM hydrogels can be used as an alternative scaffold for human cerebral organoid formation, and may be further optimized for improved organoid growth by further improving protein retention other than collagen after decellularization.”

Also, the conclusion was adopted to reflect the implications of the remaining collagen content(L 595-601):

In this study, we showed that porcine B-ECM hydrogels could be produced from native adult porcine brain, with few host cells remaining. During DC and hydrogel processing, several functional proteins were lost, however, this did not negatively impact organoid utility. After DC, the B-ECM consisted mainly of different collagen types, which is beneficial in terms of hydrogel stability, but may not have the same beneficial functional effect as if more functional, brain-specific proteins were retained. Hence, the process described here can be further optimized to reduce extraction losses and increase the content of functional proteins in the hydrogel.

*There are also a few grammatical errors like the use of the word microvascular, which is an adjective, as a noun.*

Thank you for your careful observations. We have changed the word “microvascular” to “microvasculature” (L 526). Also, additional small grammatical errors were corrected throughout the manuscript, with the help of a grammar software.

*Reviewer #2: All of my previous concerns were well addressed. The revised manuscript is well deserved to be published in PLoS One.*
